# An exploratory GIS-based method to identify and characterise landscapes with an elevated epidemiological risk of Rhodesian human African trypanosomiasis

**DOI:** 10.1186/1471-2334-12-316

**Published:** 2012-11-21

**Authors:** Nicola A Wardrop, Eric M Fèvre, Peter M Atkinson, Abbas SL Kakembo, Susan C Welburn

**Affiliations:** 1 Geography and Environment, University of Southampton, Highfield Campus, Southampton, SO17 1BJ, United Kingdom; 2 School of Biological Sciences, University of Edinburgh, Ashworth Laboratories, Kings Buildings, West Mains Road, Edinburgh, EH9 3JT, United Kingdom; 3 Ministry of Health, Department of National Disease Control, Kampala, Uganda; 4 School of Biomedical Sciences, University of Edinburgh, 49 Little France Crescent, Edinburgh, EH16 4SB, United Kingdom

**Keywords:** *Trypanosoma brucei rhodesiense*, Tsetse, *Glossina fuscipes*, GIS, Spatial epidemiology, Landscape epidemiology, Sleeping sickness, Disease ecology

## Abstract

**Background:**

Specific land cover types and activities have been correlated with *Trypanosoma brucei rhodesiense* distributions, indicating the importance of landscape for epidemiological risk. However, methods proposed to identify specific areas with elevated epidemiological risk (i.e. where transmission is more likely to occur) tend to be costly and time consuming. This paper proposes an exploratory spatial analysis using geo-referenced human African trypanosomiasis (HAT) cases and matched controls from Serere hospital, Uganda (December 1998 to November 2002) to identify areas with an elevated epidemiological risk of HAT.

**Methods:**

Buffers 3 km from each case and control were used to represent areas in which village inhabitants would carry out their daily activities. It was hypothesised that the selection of areas where several case village buffers overlapped would enable the identification of locations with increased risk of HAT transmission, as these areas were more likely to be frequented by HAT cases in several surrounding villages. The landscape within these overlap areas should more closely relate to the environment in which transmission occurs as opposed to using the full buffer areas. The analysis was carried out for each of four annual periods, for both cases and controls, using a series of threshold values (number of overlapping buffers), including a threshold of one, which represented the benchmark (e.g. use of the full buffer area as opposed to the overlap areas).

**Results:**

A greater proportion of the overlap areas for cases consisted of seasonally flooding grassland and lake fringe swamp, than the control overlap areas, correlating well with the preferred habitat of the predominant tsetse species within the study area (*Glossina fuscipes fuscipes*). The use of overlap areas also resulted in a greater difference between case and control landscapes, when compared with the benchmark (using the full buffer area).

**Conclusions:**

These results indicate that the overlap analysis has enabled the selection of areas more likely to represent epidemiological risk zones than similar analyses using full buffer areas. The identification of potential epidemiological risk zones using this method requires fewer data than other proposed methods and further development may provide vital information for the targeting of control measures.

## Background

The environmental landscape within an area is a significant factor in determining the spatial distribution of many different disease vectors, reservoirs, intermediate hosts and parasites and, thus, also the spatial distribution of a variety of diseases, including human African trypanosomiasis (HAT; also known as sleeping sickness). These correlations can be quantified and described to allow greater understanding and highlight areas with potentially higher risks of vector or reservoir presence, disease transmission, or both [1-3]. *Trypanosoma brucei rhodesiense*, the vector transmitted parasite subspecies which causes the fatal disease Rhodesian HAT, is reliant on the availability of suitable habitat and environmental conditions for the tsetse vector (*Glossina* spp.). Due to this association with particular types of land cover, HAT (and tsetse) distributions can be correlated with landscape information that captures the distribution of potential tsetse habitats. An increased risk of Rhodesian HAT in areas close to ‘long vegetation swamp’ habitats has been detected in two recent studies [4,5]. Several other studies have examined tsetse populations and risk of Gambian HAT (caused by *Trypanosoma brucei gambiense*) in relation to the presence of particular crop types (such as coffee or cocoa) [6,7], the level of human land use, disturbance of vegetation and also human movement patterns [8-10].

The recent spread of Rhodesian HAT in Uganda, the only country which sustains active transmission of both *T*. *b*. *rhodesiense* and *T*. *b*. *gambiense*[11], has led to increasing concern over a potential future overlap of the two forms of the disease [12]. The north-west spread of *T*. *b*. *rhodesiense*, which has been attributed to the movement of infected livestock (the main reservoir of the parasite in Uganda) from endemic areas, has brought areas of transmission of the two forms within 150 km of one another [11-15]. Treatment protocols differ between the two forms of HAT and the current diagnostic methods available in affected areas of Uganda are not suitable for definitive sub-species differentiation. Thus, a future overlap may severely compromise treatment and increase the likelihood of treatment failures. In light of this recent spread of Rhodesian HAT, there is an urgent need for evidence-based, spatially focused control measures.

Rhodesian HAT occurs in poor, remote, rural areas with low human population densities and evidence suggests that the majority of *T*. *b*. *rhodesiense* infections are acquired outside of the village of residence [5,16,17]. Specific activities have been implicated in Rhodesian HAT acquisition such as watering livestock and collecting water or firewood, implicating the landscape profile of areas surrounding the village of residence in epidemiological risk [16,17]. Despite the apparent advantages of spatially targeted disease control within individual HAT foci, few attempts have been made to identify the specific locations that HAT cases acquire their infections. The majority of research focuses on the village or household of residence as the spatial entity to which epidemiological data is attached, although the analysis of this type of data will not allow the identification of areas with an elevated epidemiological risk. Laveissière *et al*. [18] proposed the use of entomological data (fly density, age and blood meals) to calculate an epidemiological risk index relating to the density of vectors and the amount of human-tsetse contact. However, the entomological surveys required for this risk index can be costly and time consuming. More recently, the identification of high risk areas for *T*. *b*. *gambiense* transmission, to allow the implementation of targeted tsetse control, was carried out by Courtin *et al*. [10] by tracking the movements of individuals (HAT cases and controls) and characterising the epidemiological risk of different sites and activities. Another recent study (focusing on Rhodesian HAT in Uganda) investigated the significance of the proportion of different sized buffer zones (circular zones, of defined radius, centred on a point of interest) surrounding homesteads that intersected with areas of wetland for HAT acquisition. It was found that areas of wetland within 500 m to 3 km of homesteads significantly increased the risk of Rhodesian HAT with the highest significance observed between 800 and 900 m [5].

The significance of wetland areas up to 3 km from the homestead for the risk of Rhodesian HAT indicates that transmission may occur up to 3 km away from the homestead [5]. In addition, the average distance of daily short-distance trips (e.g. to work or to fetch water) for village residents in Uganda (the predominant population group in *T*. *b*. *rhodesiense* endemic areas) has been estimated to range from approximately 2 km for low income households to 4 km for high income households [19], reinforcing the hypothesis that HAT transmission occurs within approximately 3 km of the homestead. Using geo-referenced epidemiological data (i.e. data that can provide spatial information on where HAT patients live), it is possible to identify the areas in which patients will normally carry out their daily activities by creating a buffer (circular zone) around their homestead or village of residence. These “daily activity areas” can be used to represent the area in which HAT acquisition most likely occurred based on the hypothesis that transmission normally occurs within 3 km of the homestead. It also follows that the areas in which a large number of HAT patient’s daily activity areas overlap may constitute areas of elevated epidemiological risk. These are areas that individuals from a number of neighbouring villages visit regularly, with landscape features that promote a high level of interaction between tsetse, livestock reservoirs (mainly cattle) and humans, thus, encouraging a high intensity of HAT transmission. Areas with an elevated epidemiological risk should be considered as priority areas for HAT control activities, including tsetse control and livestock based interventions.

The current research aims to provide a starting point for the identification of locations with elevated transmission of Rhodesian HAT (due to high levels of contact between humans and tsetse) in comparison to other areas by combining previous findings with epidemiological and environmental data. The exploratory approach discussed above was used, combining geo-referenced Rhodesian HAT patient records (and matched controls) and information on the average daily distances travelled to identify areas with an elevated epidemiological risk in Soroti and Serere districts, Uganda, over a four year period. A classified land cover map for the area was created using Landsat Enhanced Thematic Mapper Plus (ETM+) imagery to allow characterisation of the landscape profiles within areas of high epidemiological risk. Utilising case records geo-located to the village of residence to identify potential high transmission areas, the costs associated with the types of studies discussed above (collecting entomological data or human movement data) can be alleviated. The elevated epidemiological risk areas identified in this manner may provide a starting point for the spatial targeting of tsetse trapping activities in the study area, and can also provide additional information on the landscape profiles conducive to intense transmission of Rhodesian HAT.

## Methods

### Study site

Soroti and Serere (which split from Soroti district in 2010) districts, which cover approximately 3,370 km^2^, are located in the Eastern region of Uganda, bordering Lake Kyoga [20]. Lake Kyoga is a large, shallow lake (maximum depth of 5.7 m) and much of the surrounding land, including parts of Soroti and Serere districts, is covered with a network of rivers, streams and swamps [21]. The population within these two districts during the 2002 national census (i.e. when the area was considered as a single district) was approximately 370,000 [22] and the predominant economic activities are subsistence farming and fishing (in areas in close proximity to the lake) [20]. From 1998 to 2002, HAT affected predominantly the area now recognised as Serere district, which is to the south of Soroti district, and is surrounded on three sides by Lake Kyoga. *Glossina fuscipes fuscipes* is the predominant vector of HAT within the study area, and its preferred habitat consists of riverine vegetation.

### Human African trypanosomiasis data

A matched case–control study design was used; passively detected Rhodesian HAT case records (including details of patient’s age, sex, date of admission and village of residence) from Serere hospital from December 1998 to November 2002 were obtained and matched to suitable controls (also from Serere hospital patient records, to avoid spatial bias). At the time of data collection, Serere Hospital was the only facility trained and equipped to diagnose and treat HAT serving the population of Soroti district. One control was matched to each case based on age group (<1, 1–9, 10–14, 15–19, 20–49, 50–64 and ≥65 years), sex and month of admission to ensure the controls adequately represented the entire population from which the HAT cases came [23]. Patients with a primary diagnosis of a vector-borne disease were excluded to prevent spatial bias in the results, which may arise due to similarities between tsetse habitat and other vector habitats. No patient identifiable information was recorded to maintain patient confidentiality and to adhere to the International Ethical Guidelines for Biomedical Research Involving Human Subjects. The use of these data was approved by the University of Edinburgh Research Ethics Committee.

The central point of the village of residence for all cases and controls was geo-referenced using a hand-held global positioning system (GPS; Garmin, Olathe, KS). Part of these data has previously been published; further information regarding their acquisition can be found in Fèvre *et al*. [13]. The dataset was stratified annually to allow the separate analysis of each year and illustrate any temporal patterns in the observed relationships. The annual periods for analysis ran from December to November and, for clarity, are referred to as the first (December 1998 to November 1999), second (December 1999 to November 2000), third (December 2000 to November 2001) and fourth (December 2001 to November 2002) annual periods.

### Land cover classification

Three level 1 Landsat ETM+ images (level 1 T; path 171, row 59; radiometrically and geometrically corrected prior to distribution) were selected from 27^th^ January, 17^th^ April and 27^th^ November 2001, corresponding to the dry season, beginning of the long rains and end of the short rains, respectively. Atmospheric correction for each of the Landsat images was carried out using ATCOR-2 (Atmospheric & Topographic Correction for Small FOV Satellite Images; ReSe Applications Schläpfer, Switzerland; [24]). During atmospheric correction, the image units were converted from digital numbers to reflectance. The normalised difference vegetation index (NDVI; a measure of the amount of green vegetation) was calculated for each image using the red and near-infrared wavebands of a Landsat ETM+ image using the following formula: NDVI = (near-infrared – red)/(near-infrared + red) in the eCognition software Version 4.2 (Definiens, Munich, Germany) [25].

Ground-based training data were collected using a handheld GPS in 2001 and using fine spatial resolution Quick Bird imagery available in Google Earth (Google™, Mountain View, CA) for land cover classes which were easily identifiable and were not likely to change over time (see Table 1 for the classes obtained). A random 20% sample of the training polygons was selected for accuracy assessment, with the remaining 80% being used to train the classification. Image segmentation and classification were carried out using the eCognition software. Segmentation was used to produce homogeneous objects for classification using a scale parameter of 5 (determines the size of the resulting objects), and homogeneity criteria with 90% emphasis on spectral homogeneity and 10% shape, and 50% for both compactness and smoothness.

**Table 1:** Land cover class descriptions

**Land cover class**	**Description**
Crops, agricultural and open savannah	Agricultural land or grassland with occasional trees/bushes (not seasonally flooding)
Woodland and dense savannah	Grassland with dense trees/bushes (not seasonally flooding) and patches of woodland
Built up and bare ground	Towns or villages with high building density or bare murrum or mud
Open water	Areas of open water
Lake fringe swamps	Lake edges with a high density of papyrus, water hyacinth and water lilies
Seasonally flooded grassland	Savannah which floods during the wet season, with occasional trees/bushes

**Figure 1:**
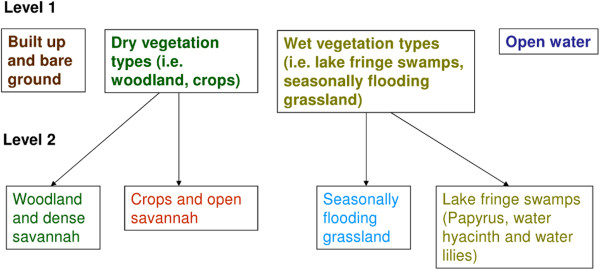
**Land cover class hierarchy showing super-classes (level 1) and sub-classes (level 2)**.

The classification was performed in two steps (an initial classification and a sub-classification), as shown in Figure 1. For the level 1 classification, open water was classified using a threshold selected by visual interpretation (Table 2, level 1 classification). A nearest neighbour algorithm was used to classify the remaining level 1 classes (built up and bare ground, dry vegetation types and wet vegetation types; the image features used are detailed in Table 2, level 1 classification). Sub-classification of the dry vegetation types (into woodland and dense savannah and crops and open savannah), and the wet vegetation types (into seasonally flooding grassland and lake fringe swamps) was carried out using nearest neighbour algorithms (the image features used are detailed in Table 2, level 2 classification). An accuracy assessment of the land cover classification was carried out by creating an error matrix and calculating producer’s and user’s accuracies for each class, plus an overall accuracy value. The open water class was excluded from the accuracy assessment as the threshold selection was not based on training data.

**Table 2:** Image features used for level 1 and level 2 classifications

	**Classes**	**Features**	**Image**	**Notes**
**Level 1 classification**	Open water	Object mean band 4 (near infrared): threshold of 5.5%	April	Light in band 4 absorbed by water
	Built up & bare ground; wet vegetation types; dry vegetation types	Object mean band 3 (red)	April	Vegetation type discrimination
		Object mean band 2 (green)	November	Identification of healthy vegetation
		Object mean band 3	November	Vegetation type discrimination
		Object mean band 5 (mid infrared)	November	Vegetation and soil moisture content
		Object mean band 7 (mid infrared)	November	Identification of built up areas and bare ground
		Object length/width		
		NDVI difference, January to November		Differentiation of vegetation types
**Level 2 classification**	Woodland & dense savannah*; crops & open savannah*	Object mean band 2	April	Related to healthy vegetation, biomass, plant type or vegetation moisture content
		Object mean band 3	April	
		Object mean band 4	April	
		Object mean band 5	April	
		NDVI difference, January to April, January to November and April to November		Differentiation of vegetation types
	Seasonally flooding grassland**; lake fringe swamps**	Object mean band 4	November	Identification of vegetation and soil moisture content, biomass, plant vigour and water
		Object mean band 5	November	

All classifications used a nearest neighbour algorithm with the exception of open water, which utilised a threshold [26,27].

*Obtained using sub-classifications of the “dry vegetation types” class.

**Obtained using sub-classifications of the “wet vegetation types” class.

### Identification and characterisation of epidemiological risk zones

**Figure 2:**
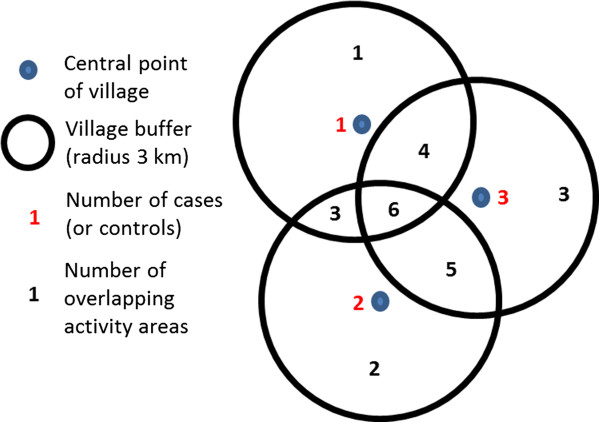
**Schematic diagram showing buffer areas surrounding village centres and the calculation of number of overlapping activity areas**.

The geo-referenced case–control data were visualised using ArcMap 9.1 (ESRI, Redlands, CA). Circular buffers of 3 km radii were created around the village centroid for each case and control (a circular zone extending 3 km from the central point of the village, see Figure 2 for an illustration); these buffers were representative of the average distance that village inhabitants walk from their village on a daily basis (for activities such as fetching water or firewood and watering livestock) and were used to represent the daily areas of mobility (referred to as ‘daily activity areas’) for cases and controls. A spatial grid was created over the entire study area, with a 500 m by 500 m cell size. The number of daily activity areas for cases which overlapped within each grid cell was calculated to give the number of intersecting daily activity areas. This was carried out for each of the four annual periods and was repeated for the control daily activity areas.

Similar, more traditional methods such as kernel density estimation (KDE; where a weighted average is calculated within a spatially moving window [28]) do not capture fully the desired effect. Weighted KDE produces a higher intensity at village centres where a lot of cases have occurred and lower intensity in areas outside villages where transmission is more likely to occur, due to the weighting used; more weight is generally given to values in the centre of the moving window than those on the periphery. KDE methods are used to give a smooth representation of the intensity of a point process [29]. As HAT transmission normally occurs outside of the village, transmission zones may not be represented accurately by village centroids and, therefore, weighted KDE will not adequately identify areas which may be considered as epidemiological risk zones. The methods presented here give results comparable to those which may be obtained using an un-weighted kernel density smoothing algorithm (a flat kernel, where equal weight is given to all values within the moving window). As seen in Figure 2, areas of overlap between neighbouring village’s buffer zones allow the identification of areas further away from the village centre, as opposed to a weighted KDE analysis.

#### Defining overlap zones

The number of intersecting daily activity areas within grid cells was used to define “overlap zones” for both controls and cases, for each annual period. These were created for a range of threshold values (thresholds were based on the number of intersecting daily activity areas); all grid cells with a value equal to, or greater than, the threshold in question were selected to create the overlap zone relating to that threshold. The thresholds used ranged from one (i.e. the grid cell contains one daily activity area) to 30 (i.e. the grid cell contains 30 overlapping daily activity areas). The range of thresholds was used to demonstrate changes in the overlap zones created using increasing numbers of intersecting daily activity areas. The threshold value of one provided a specific outcome in terms of buffer area that was very useful as a benchmark comparison; it relates to a full buffer surrounding each village, as opposed to the area of intersecting buffers. Thus, we were able to compare the use of intersecting buffer areas (the novel approach in this paper) with a full buffer, equivalent to a standard unweighted kernel density estimation approach.

#### Characterising overlap zones

The areas of different land cover classes within the overlap zones (relating to the full range of threshold values) were calculated and expressed as a proportion of the overall zone area for each annual period, for both cases and controls. The *z*-test for two proportions was used to assess the significance of the difference in proportions of the various land cover types within overlap zones for cases and controls, at each threshold. Plots demonstrating the proportion of land cover classes within the overlap zones from each threshold value were produced for land cover classes which demonstrated consistent significant differences between case and control overlap zones. These plots were used to illustrate the change in land cover within zones with increasing thresholds (i.e. with a higher number of overlapping daily activity areas) and the difference between case and control zones.

#### Defining and characterising high overlap areas

Grid cells in the 99^th^ percentile based on the number of intersecting daily activity areas (the 1% of grid cells with the highest number of intersecting buffers) were defined as being areas with a high number of overlapping daily activity areas (these will be referred to as ‘high overlap areas’), to provide an illustration of temporal changes in the characteristics of high overlap areas. The analysis was also carried out for the control daily activity areas for each of the four annual periods to highlight differences in the location and characteristics of high overlap areas for cases and controls, over the four year period. The high overlap areas for cases may be considered as areas with an elevated epidemiological risk. The areas of different land cover classes within each of the high overlap areas were calculated and expressed as a proportion of the overall high overlap area for each of the four annual periods, for both cases and controls. In addition, the average elevation within the high overlap areas was extracted [30].

The *z*-test for two proportions was used to assess the significance of the difference in proportions of the various land cover types within high overlap areas for cases and controls. Open water and lake-fringe swamps were not included in this section of the analysis due to the predominance of high overlap areas at the selected threshold values with no intersections with these land cover types. Additionally, a *t*-test was used to assess the significance of the difference between mean elevations for case and control high overlap areas during each of the annual periods. Percentage component bar charts created in Microsoft® Excel were used to highlight the difference in landscape profiles for case and control high overlap areas and to assess any temporal changes. A line plot was also used to demonstrate changes in the mean elevation within the case and control high overlap areas over time.

## Results

### Supervised object-based classification

The overall accuracy for the land cover classification was 86%, with producer’s and user’s accuracies of over 70% for all classes except crops and open savannah (producer’s accuracy = 69%, user’s accuracy = 47%). The class crops and open savannah was not thought to be a significant tsetse habitat within the study area and so the lower accuracy for this class was not problematic, although it may have resulted in lower proportions of the other classes of relevance as potential tsetse habitat. Within the study area (a subset of the entire classified image), the predominant land cover classes were crops and open savannah (31.4% of the study area), followed by open water and woodland and dense savannah (both 17.9%). Seasonally flooding grassland accounted for 14.3%, lake-fringe swamps 11% and built up and bare ground was the least common land cover class (7.5%).

### Exploratory analysis

A total of 258 Rhodesian HAT cases resident within Soroti district were diagnosed at Serere hospital during the study period (December 1998 to November 2002). Eighteen of these were detected during active surveillance activities and, thus, were excluded from the analysis. In addition, suitable matched controls could not be identified for seven of the cases, and the unmatched cases were excluded from the analysis. A total of 58 cases were analysed in the first annual period, 52 in the second annual period, 44 in the third annual period and 79 in the fourth annual period (each with the same numbers of matched controls).

**Figure 3:**
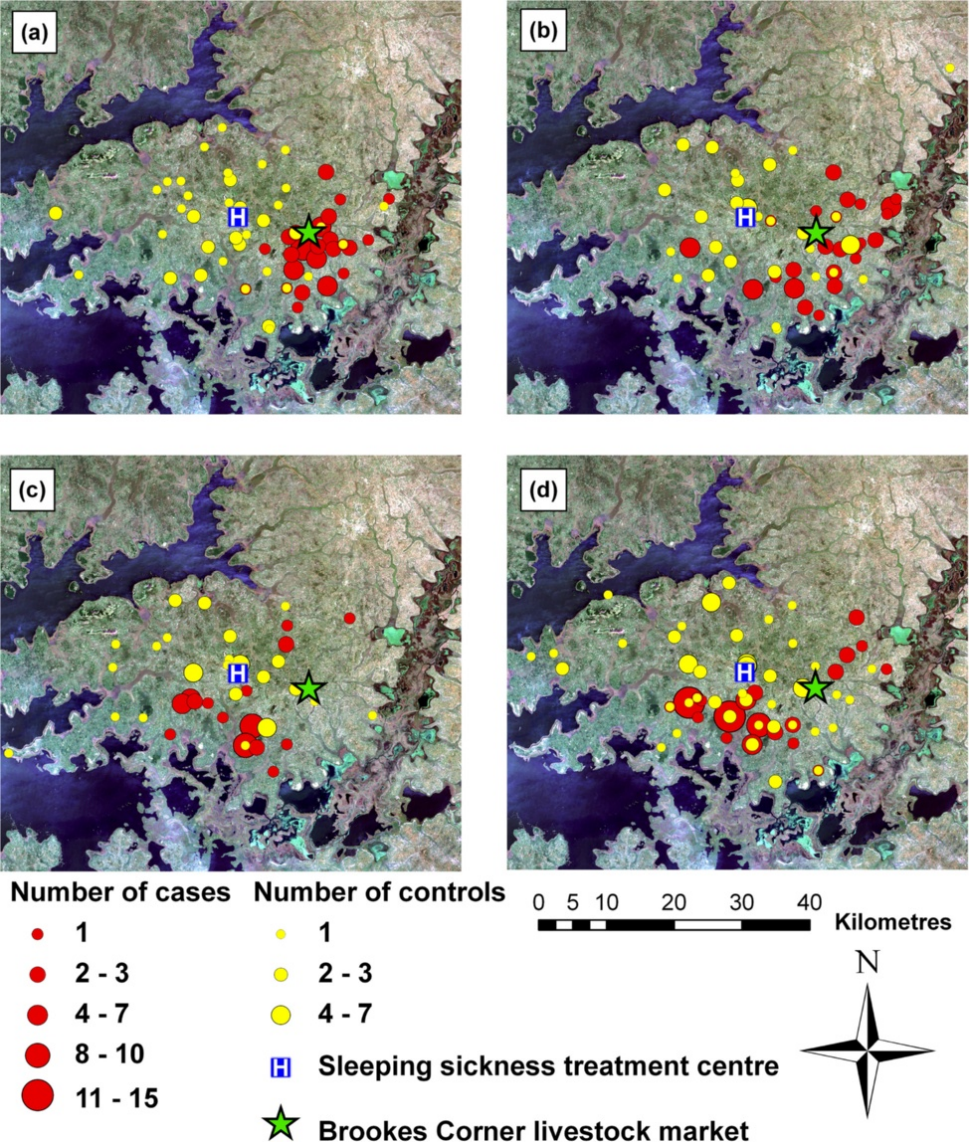
**True colour Landsat ETM+ composite of study area.** True colour Landsat ETM+ composite of study area showing distribution of cases and controls (as counts for each location) in the first (**a**), second (**b**), third (**c**) and fourth (**d**) annual periods, with HAT treatment centre and Brookes Corner livestock market.

The spatial distribution of cases within the study area varied through the study period (Figures 3a to 3d). Between December 1998 and November 1999, the cases were primarily located in close proximity to Brookes Corner livestock market (Figure 3a). The parasite has previously been demonstrated to have been introduced to the study area via the trade of untreated cattle at this market [13]. In contrast, the controls were evenly distributed across the study area, with no apparent clustering. Over the subsequent years, the spatial distribution of controls remained evenly dispersed across the study area with no directional movement, while the distribution of cases changed, with a directional movement away from the livestock market (Figures 3b, 3c and 3d) as has been previously reported [13]. 

### Identification and characterisation of epidemiological risk zones

**Figure 4:**
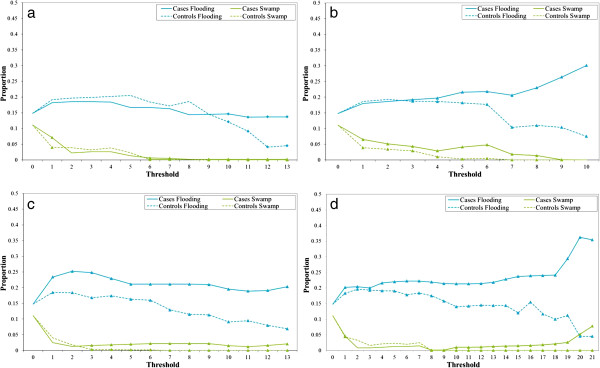
**Overlap zone landscape profiles at different threshold values for each of the four annual periods.** Proportion of overlap zones consisting of seasonally flooding grassland (Flooding) and lake fringe swamp (Swamp) at different threshold values for the first (**a**), second (**b**), third (**c**) and fourth (**d**) annual periods. Threshold values were based on the number of intersecting daily activity areas and a threshold of one represents the benchmark, using the full buffer area rather than overlap areas.

The proportions of overlap zones which consisted of lake fringe swamp and seasonally flooding grassland were consistently significantly different between case and control overlap zones, across increasing threshold values and through the four annual periods (Figure 4a to d). Case overlap zones contained a higher proportion of both lake fringe swamp and seasonally flooding grassland in comparison to control overlap zones, with statistical significance (p<0.05) for the majority of threshold values. A larger difference between cases and controls can be observed when comparing the overlap zones (thresholds greater than one) with the benchmark (threshold of one; using the whole buffer area rather than overlap areas). In addition, during the first and second annual periods, the benchmark analysis demonstrated higher proportions of seasonally flooding grassland for control buffers than case buffers, and in the third annual period, a higher proportion of lake fringe swamp was present in control buffers than case buffer. These relationships, observed using the full buffer areas rather than overlap zones, are the reverse of those detected consistently across the four annual periods and at increasing thresholds. There were no consistent patterns for the crops, agriculture and open savannah, built up and bare ground or open water land cover classes (data not shown). The woodland and dense savannah land cover classes tended to occupy a greater proportion of the control overlap zones than case high overlap zones, although this was mainly non-significant (data not shown).

### Identification of high overlap zones

**Figure 5:**
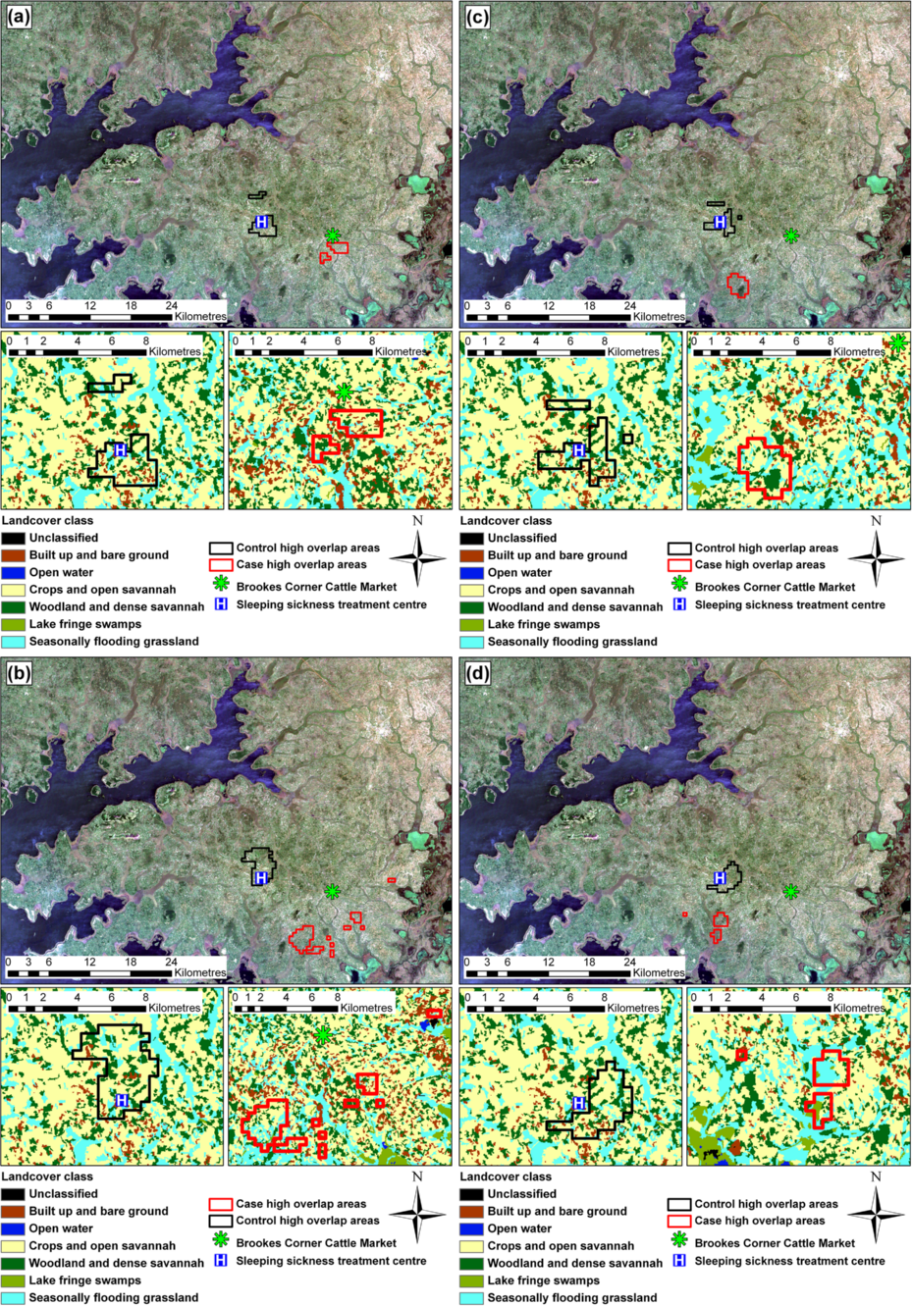
**Case and control high overlap areas.** True colour Landsat ETM+ composite of study area highlighting case (red) and control (black) high overlap areas in the first (**a**), second (**b**), third (**c**) and fourth (**d**) annual periods, also with close up images of high overlap areas with land cover classes.

Several high overlap areas were identified for each of the four annual periods using grid cells in the 99^th^ percentile, based on the number of intersecting daily activity areas (see Figures 5a - d for maps highlighting the case and control high overlap areas). The high overlap areas for the controls were distinct from those of the cases. For each time period, control high overlap areas were located within close proximity to the HAT treatment centre (Serere Hospital), and no directional trend was evident across the four annual periods. The high overlap areas for cases (elevated epidemiological risk zones) in the first annual period were located close to Brookes Corner livestock market, which has previously been identified as the original source of the disease within the study area [13]. The high overlap areas for the cases moved from the original location (close to Brookes Corner livestock market) in a south-westerly direction in subsequent years. 

### Characterisation of epidemiological risk zones

During the first annual period the proportion of built up and bare ground, crops and open savannah and seasonally flooding grassland were not significantly different between the high overlap areas for cases and controls (see Table 3), although the case high overlap areas had a significantly lower proportion of woodland and dense savannah than control high overlap areas (*p* < 0.001). The same significant difference was seen in the second and final annual periods (*p* < 0.001), although in the third period the difference was reversed and the proportion of woodland and dense savannah was higher for the high overlap areas of cases than controls (*p* < 0.001).

**Table 3:** Land cover profiles for high overlap areas, and mean elevation for the four annual periods

**Annual period**		**Proportion of high overlap area classified as:**				**Mean elevation (metres)**
		**Built up & bare ground**	**Crops & open savannah**	**Seasonally flooding grassland**	**Woodland & dense savannah (2-tailed)**	
Dec 1998 - Nov 1999	Cases	10.48%	61.05%	10.09%	18.38%	1098.27
	Controls	10.19%	55.21%	12.15%	22.44%	1095.37
	Difference	0.29%	5.84%	−2.06%	−4.07%	2.90
	*p*-value	0.72	>0.99	>0.99	<0.001	>0.99
Dec 1999 - Nov 2000	Cases	6.96%	48.86%	26.38%	17.13%	1066.31
	Controls	4.54%	59.61%	11.04%	24.81%	1100.10
	Difference	2.42%	−10.76%	15.34%	−7.67%	−33.79
	*p*-value	>0.99	<0.001	<0.001	<0.001	<0.001
Dec 2000 - Nov 2001	Cases	1.54%	52.66%	17.14%	28.66%	1058.35
	Controls	7.88%	59.56%	9.53%	23.03%	1099.74
	Difference	−6.34%	−6.90%	7.61%	5.64%	−41.39
	*p*-value	<0.001	<0.001	<0.001	<0.001	<0.001
Dec 2001 - Nov 2002	Cases	0.90%	33.68%	41.60%	14.06%	1047.82
	Controls	5.81%	60.84%	10.02%	23.34%	1093.86
	Difference	−4.91%	−27.15%	31.58%	−9.27%	−46.04
	*p*-value	<0.001	<0.001	<0.001	<0.001	<0.001

*P*-values shown represent the significance of the difference in proportion (for land cover classes) from a *z*-test, or difference in mean (for elevation) from a *t*-test. The alternative hypotheses for the proportion of built up and bare ground and crops and open savannah classes and mean elevation were that the proportion or mean for case high overlap areas would be lower than for control high overlap areas (one-tailed test). The alternative hypothesis for the seasonally flooding grassland class was that the proportion would be higher for case high overlap areas than control high overlap areas (one-tailed test). The alternative hypothesis for the woodland and dense savannah class was that the difference in proportions would not be zero (two-tailed test).

**Figure 6:**
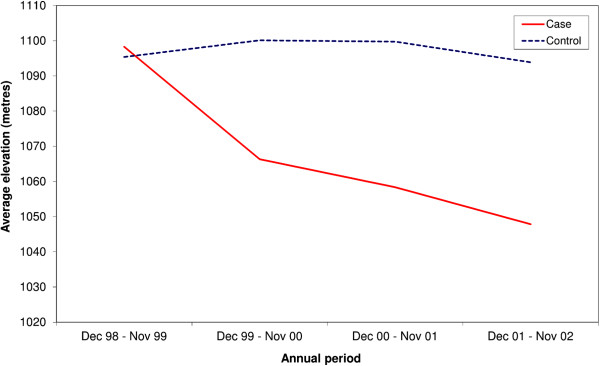
**Average elevation within case and control high overlap areas, over the four annual periods**.

From the second annual period onwards, the proportion of high overlap areas classified as crops and open savannah was significantly lower for cases than controls (*p* < 0.001 for each annual period), and the proportion classified as seasonally flooding grassland was significantly higher for cases than controls (*p* < 0.001 for each annual period). The proportion of built up and bare ground was significantly lower in high overlap areas for cases than controls in the two final annual periods (*p* < 0.001 for both annual periods). Additionally, the mean elevations within the high overlap zones were significantly lower for cases than controls for the second, third and fourth annual periods (*p* < 0.001 for each of these three periods; see Table 3 and Figure 6).

**Figure 7:**
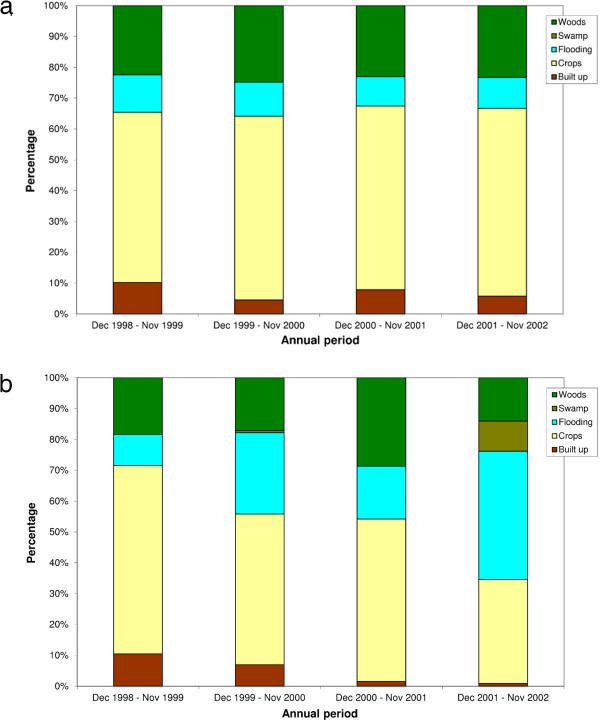
**Land cover profile within high overlap areas.** Land cover profile within high overlap areas for cases (**a**) and controls (**b**) over the four annual periods.

From Table 3 and Figure 7 b, the changing landscape over time within the epidemiological risk zones can be observed. The proportion of high overlap areas that were classified as crops and open savannah decreased from 62% to 34%; the proportion classified as built up and bare ground decreased from 10% to 1%, and the proportion classified as seasonally flooding grassland increased from 10% to 41%. In comparison, the landscape seen in high overlap areas for controls remained relatively constant over time, with a predominance of the crops and open savannah land cover class (see Figure 7 a). The average elevation within the epidemiological risk zones also demonstrated a temporal trend, with a decreasing mean elevation over time (decreasing from 1098 m during the first annual period, to 1048 m in the fourth annual period; see Figure 6), while the average elevation for the control high overlap areas remained relatively constant over the study period.

## Discussion

The investigation of areas with elevated risk of HAT transmission is difficult as infection normally occurs outside of the village of residence in areas where humans come into contact with tsetse [16,17]. The location of a HAT patient’s village of residence or homestead does not provide sufficient information to identify the areas in which an elevated epidemiological risk occurs or the landscape features contributing to this increased infection risk. The methods which have previously been used to identify high transmission risk areas (e.g. the use of entomological sampling or the tracking of human movements) are time consuming and can be costly [10,18]. Here, a novel method has been explored, utilising knowledge of the distance outside of the village of residence travelled by village inhabitants on an average day to identify the areas which are likely to support elevated HAT transmission. The data acquisition required for this method is less time and resource intensive than previous methods, allowing the identification of potential epidemiological risk areas with minimal field-based surveys. Consistent differences between the overlap zones of cases and controls were detected and several epidemiological risk zones identified; these areas are likely to be frequented by the residents of a number of surrounding villages (i.e. to water and graze livestock or collect firewood), and due to their particular landscapes and environmental conditions, may promote a high level of interaction between tsetse vectors, livestock and humans.

The application of buffer analysis in spatial epidemiology is not uncommon, and is typically used to include landscape features surrounding the home or village of residence in an analysis (e.g., [4,31]), particularly in cases where disease transmission is expected to occur outside of the home or village. However, it is unlikely that the landscape within the full buffer area contributes to the risk of disease transmission, particularly in the case of Rhodesian HAT, where transmission tends to occur in localised areas which promote increased interaction between humans, livestock and tsetse. The use of full buffer areas in these situations will dilute attempts to delineate epidemiological risk areas and will weaken correlations between the locations of cases and the occurrence of specific landscape features (e.g. specific types of land cover). By identifying areas which are within 3 km of several HAT patients’ villages (e.g. overlap areas) rather than using the full buffer areas, it is possible to select portions of the buffer which are more likely to represent an increased epidemiological risk of HAT.

An analysis of the landscape within overlap zones using a range of thresholds (based on the number of overlapping daily activity areas) illustrated consistency (across the four annual periods and with increasing threshold values) in the differences between the overlap zones for cases and controls with respect to the proportions occupied by seasonally flooding grassland and lake fringe swamp. The significantly higher proportions of these land cover classes in case overlap zones than control overlap zones correlates with the habitat requirements of *Glossina fuscipes fuscipes*, the primary vector within the study area (prefers riverine vegetation). These results indicate that the landscape in areas which are likely to have been frequented by HAT patients differs from the landscape in areas likely to have been frequented by controls, and is more likely to support *Glossina* spp. populations and, therefore, transmission of HAT. The difference in landscape between cases and controls was smaller using the full buffer areas, and in some cases the consistent trends described above were reversed. This indicates that the benchmark situation may not have adequately targeted the epidemiological risk areas and the specific landscape profiles within the selected areas may have been diluted.

Using the 99^th^ percentile for the selection of high overlap zones, a distinct difference can be observed between the case and control zones; the high overlap areas for cases (epidemiological risk zones) moved gradually over the study period, but the high overlap areas for controls remained relatively static and close to Serere hospital. A clear temporal trend in the landscape can be observed within these high overlap zones over the four annual periods. Within the first annual period, there was no significant difference in the proportional coverage of crops and open savannah or seasonally flooding grassland or the elevation between case and control high overlap areas. No significant difference was observed for the proportion of high overlap areas that was built up and bare ground for the first or second annual periods. In subsequent years, however, the differences between case and control high overlap areas became statistically significant; there was a significantly lower proportion of the land cover classes built up and bare ground and crops and open savannah in the case high overlap zones and a significantly larger proportion of seasonally flooding grassland. In addition, the average elevation was significantly lower in case high overlap areas than controls in the second, third and fourth annual periods. For the woodland and dense savannah land cover classes, the proportion of high overlap area was larger for controls than cases for all annual periods, except for the third annual period where this pattern was reversed.

The temporal movement of the potential high transmission zones and the changing landscape profiles within these areas reflects the spatial dispersal of the disease outwards from the point of initial introduction (the livestock market) over time [13,32]. The landscapes within the potential high transmission zones, particularly in the third and fourth annual periods, may constitute areas with a higher risk of *T*. *b*. *rhodesiense* transmission due their greater suitability for vector populations, combined with an increased amount of interaction between humans, livestock and tsetse. As the potential high transmission zones were, by definition, areas outside the village, the human population density of the areas should be low. The low population density and a lower level of human disturbance (due to less agricultural activity as evidenced by a lower proportion of the land cover class crops and open savannah) results in less disturbance of potential tsetse habitat. The presence of land cover classes which provide suitable tsetse habitat (such as seasonally flooding grassland), in combination with less human disturbance may partly explain an elevated transmission risk. In addition to the characterised landscape profiles within the epidemiological risk zones, it is likely that local factors influencing the daily movement patterns of village inhabitants from surrounding villages also play a role. For example areas of seasonally flooding grassland may be used frequently for the watering of livestock for surrounding villages, thus, increasing contact between humans, livestock and tsetse flies and promoting transmission of *T*. *b rhodesiense*. The accessibility of these areas may also contribute, although it has not been possible to examine such factors using the data available.

The selection of a single threshold value to allow the observation of differences between case and control high overlap zones across each of the four annual periods is not a straightforward decision. Ideally, to allow comparability between the results for each of the four annual periods, the same threshold value would be used for each. However, the varying case (and, therefore, control) numbers in each of the annual periods, in addition to spatial heterogeneity, meant that the maximum number of overlapping daily activity areas differed for cases and controls, and for each of the four annual periods. The 99^th^ percentile was selected to give an initial illustration of the potential for this exploratory method, but future work should consider refining the threshold selection.

The information provided from this type of exploratory analysis may enable the micro-scale spatial targeting of tsetse control activities, including the employment of tsetse traps or livestock based control (treatment and restricted application [RAP] of insecticides to livestock) [33,34]. The implementation of spatially targeted vector control in areas where people may be at a greater risk of acquiring HAT (due to landscape features) may have a direct impact on transmission of *T*. *b*. *rhodesiense* to humans at a local scale, enabling a reduction in the burden of Rhodesian HAT in the most affected communities. Localised vector control along with RAP insecticide use across larger areas (e.g. districts or sub counties) may complement one another by focusing on both the interruption of local transmission cycles and the reduction of *T*. *b*. *rhodesiense* prevalence in reservoirs in a spatially hierarchical manner. The spatial targeting of traps in locations identified as having an elevated epidemiological risk has also been proposed for the control of Gambian HAT in West Africa [10].

## Conclusions

This paper has proposed and applied an exploratory method to identify and characterise the specific areas outside of villages which may present an elevated epidemiological risk of *T*. *b*. *rhodesiense* transmission to humans. This method uses a minimal number of data (passive surveillance records and geo-referenced villages for cases and controls) and, thus, future development may lead to a cost-effective method to identify potential epidemiological risk areas. A thorough understanding of the areas in which Rhodesian HAT infections are acquired could provide invaluable information for the spatial targeting of tsetse traps to high risk landscapes, planning of livestock based control activities or targeted community education. Potential future research includes the refinement and testing of this method via the incorporation of local road, track and footpath networks to establish passable routes from villages into the high overlap areas and to allow the more detailed identification of priority areas for tsetse trap deployment. The addition of finer spatial resolution landscape covariates may also allow a more detailed characterisation of the potential epidemiological risk zones.

## Competing interests

The authors declare no competing financial interests.

## Authors’ contribution

The data acquisition was conducted by EMF. The study design was conceived and data analysis performed by NAW. PMA provided expertise and assistance with land cover classification and spatial data analysis. The manuscript was written by NAW and PMA, with all authors contributing to the interpretation of results and the final manuscript.

## Pre-publication history

The pre-publication history for this paper can be accessed here:

http://www.biomedcentral.com/1471-2334/12/316/prepub
